# Ecrg4 expression and its product augurin in the choroid plexus: impact on fetal brain development, cerebrospinal fluid homeostasis and neuroprogenitor cell response to CNS injury

**DOI:** 10.1186/2045-8118-8-6

**Published:** 2011-01-18

**Authors:** Ana Maria Gonzalez, Sonia Podvin, Shuh-Yow Lin, Miles C Miller, Hannah Botfield, Wendy E Leadbeater, Andrew Roberton, Xitong Dang, Stuart E Knowling, Elena Cardenas-Galindo, John E Donahue, Edward G Stopa, Conrad E Johanson, Raul Coimbra, Brian P Eliceiri, Andrew Baird

**Affiliations:** 1 College of Medical and Dental Sciences, University of Birmingham, Edgbaston, Birmingham, B15 2TT, UK; 2 Department of Surgery, School of Medicine, University of California, San Diego, 200 West Arbor Drive, San Diego, California, 92103, USA; 3 Departments of Neurosurgery and Pathology, Rhode Island Hospital, Warren Alpert Medical School of Brown University, 593 Eddy Street, Providence, RI, 02903, USA

## Abstract

**Background:**

The content and composition of cerebrospinal fluid (CSF) is determined in large part by the choroid plexus (CP) and specifically, a specialized epithelial cell (CPe) layer that responds to, synthesizes, and transports peptide hormones into and out of CSF. Together with ventricular ependymal cells, these CPe relay homeostatic signals throughout the central nervous system (CNS) and regulate CSF hydrodynamics. One new candidate signal is augurin, a newly recognized 14 kDa protein that is encoded by *esophageal cancer related gene-4 *(*Ecrg4*), a putative tumor suppressor gene whose presence and function in normal tissues remains unexplored and enigmatic. The aim of this study was to explore whether *Ecrg4 *and its product augurin, can be implicated in CNS development and the response to CNS injury.

**Methods:**

*Ecrg4 *gene expression in CNS and peripheral tissues was studied by *in situ *hybridization and quantitative RT-PCR. Augurin, the protein encoded by *Ecrg4*, was detected by immunoblotting, immunohistochemistry and ELISA. The biological consequence of augurin over-expression was studied in a cortical stab model of rat CNS injury by intra-cerebro-ventricular injection of an adenovirus vector containing the *Ecrg4 *cDNA. The biological consequences of reduced augurin expression were evaluated by characterizing the CNS phenotype caused by *Ecrg4 *gene knockdown in developing zebrafish embryos.

**Results:**

Gene expression and immunohistochemical analyses revealed that, the CP is a major source of *Ecrg4 *in the CNS and that *Ecrg4 *mRNA is predominantly localized to choroid plexus epithelial (CPe), ventricular and central canal cells of the spinal cord. After a stab injury into the brain however, both augurin staining and *Ecrg4 *gene expression decreased precipitously. If the loss of augurin was circumvented by over-expressing *Ecrg4 in vivo*, BrdU incorporation by cells in the subependymal zone decreased. Inversely, gene knockdown of *Ecrg4 *in developing zebrafish embryos caused increased proliferation of GFAP-positive cells and induced a dose-dependent hydrocephalus-like phenotype that could be rescued by co-injection of antisense morpholinos with *Ecrg4 *mRNA.

**Conclusion:**

An unusually elevated expression of the *Ecrg4 *gene in the CP implies that its product, augurin, plays a role in CP-CSF-CNS function. The results are all consistent with a model whereby an injury-induced decrease in augurin dysinhibits target cells at the ependymal-subependymal interface. We speculate that the ability of CP and ependymal epithelium to alter the progenitor cell response to CNS injury may be mediated, in part by *Ecrg4*. If so, the canonic control of its promoter by DNA methylation may implicate epigenetic mechanisms in neuroprogenitor fate and function in the CNS.

## Background

The choroid plexus (CP) is a unique structure in the central nervous system (CNS) that is both a major source of cerebrospinal fluid (CSF) as well as small molecules, peptides and proteins that maintain overall brain health, hydrodynamics and homeostasis. The CP epithelium (CPe) either translocates these factors from the blood or synthesizes and releases them into the CSF [1]. As a specialized, yet continuous, extension of the ependymal epithelium that lines the ventricles in the brain [2], both CPe and ventricular ependymal cells share hydrodynamic and homeostatic functions in regulating CSF flow and function [3]. In addition, these epithelial cells assist in the recovery and regeneration after CNS injury [4,5]. With an ability to regulate the fate of neural stem/progenitor cells (NSPCs) that lie in the physically adjacent subventricular zone (SVZ) of the ventricular surface [6], both CPe and ependymal epithelial cells are beginning to be viewed as having a central role in CNS repair [7]. As such, trophic factors produced by these epithelial cells could have profound effects on tissue repair and regeneration in the CNS [8].

**Figure 1:**
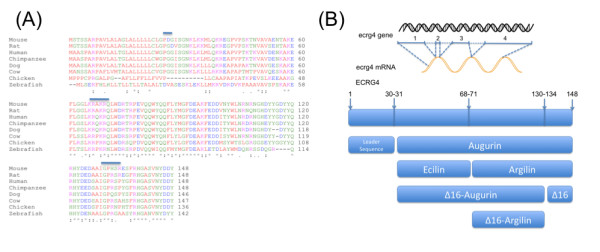
***ECRG4 *homologies and its predicted processing**: A: ECRG4 encodes conserved, potentially secreted proteins. Homologies between species of *Ecrg4 *encoded proteins are evident after the primary sequence alignment of the proteins encoded by mouse, rat, human, chimpanzee, dog, cow, chicken and zebrafish *Ecrg4*(a) gene were collated from Pubmed (nucleotide) and compared. To aid in functional comparisons, the sequences were labeled red for small amino acids, blue for acidic and magenta for basic amino acids, green for hydroxyl-, sulfhydryl, aminated or glycine amino acids. Actual homologies are presented in table 1. Consensus processing sites for (1) removal of the signal peptide, (2) processing by furin-like hormone substrates and (3) thrombin is highlighted by a line over the sequences. B: Candidate proteins generated from Ecrg4 gene expression. Eight potential protein products are predicted by algorithms to be generated from the single gene: intact *Ecrg4*(1-148); its leader sequence, augurin, argilin and ecilin and their ∆16C-terminal cleaved homologs: C∆16-augurin, C∆16-argilin and the ∆16 peptide itself.

Modern genomics has generated public databases that describe the normal distribution of gene expression in tissues, changes following experimental manipulations of cell and animal models, and alterations associated with human disease [9]. Coupled with a multitude of bio-informatic methodologies publicly available on the Internet and made available by consortia of laboratories, investigators can now mine these databases to explore the presence of unique gene sets, the distribution patterns of gene expression, and the possible existence of unique structural features in predicted gene products. For example, in 2007 Mirabeau *et al *[10] extended a hidden Markov modeling bioinformatics approach originally used by Bi *et al *[11] to search deeper into the human genome for genes that might encode novel peptide hormones. These investigators then recognized that a gene that was originally discovered in 1998 [12] and termed *esophageal cancer related gene-4 *(*Ecrg4*) had the features of a neuropeptide within its primary sequence, but no known biological function. Instead, it was widely recognized as being down-regulated in many, if not most, cancers [12-18]. Markov models of protein processing predicted that the *Ecrg4 *open reading frame (ORF) encoded at least one, if not several, potentially highly conserved secreted proteins, one of which Mirabeau *et al *[10] termed augurin (Figure 1).

**Figure 2:**
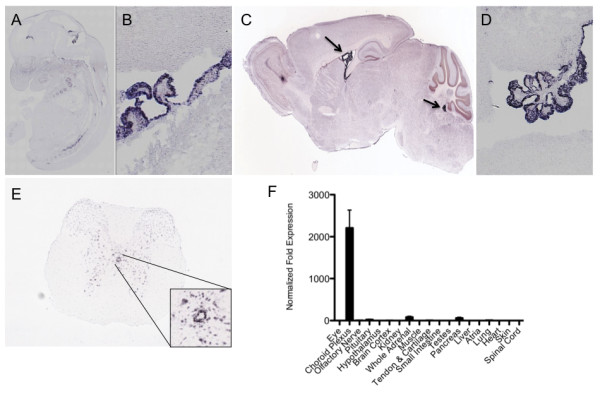
**The choroid plexus and ependyma are major sites of *Ecrg4 *expression**. A: Ecrg4 gene expression in the mouse embryo. *In situ *hybridization of mouse embryo day E14.5 were obtained from the GenePaint consortium [51]. Significantly more data is available at: http://www.genepaint.org/cgi-bin/mgrqcgi94?APPNAME=genepaint&PRGNAME=analysis_viewer&ARGUMENTS=-AQ64622747128404,-AEG,-A804,-Asetstart,-A5 B: Ecrg4 gene expression in the developing mouse brain. Higher magnification analysis of the CNS localization (from samples shown in A), shows the i*n situ *hybridization signal localizing primarily to choroid plexus and ependyma. C: Ecrg4 gene expression in the adult mouse brain. *In situ *hybridization of adult mouse brain were obtained from open source data generated by the Allen Brain Atlas [21]. Significantly more data throughout the brain are available for analyses at: http://mouse.brain-map.org/brain/1500015O10Rik/70429477/thumbnails.html?ispopup=1. D: Ecrg4 gene expression in the mouse choroid plexus and ependyma. A higher magnification analysis of the CNS localization (from samples shown in Panel C), show that the i*n situ *hybridization signal localizes primarily to choroid plexus epithelial cells and to a lesser extent ventricular ependymal cells. E: Ecrg4 gene expression in mouse central canal ependymal/epithelial cells in the spinal cord. *In situ *hybridization maps of adult mouse spinal cord were selected from open source data generated by the Allen Brain Atlas Consortium [21]. Significantly more data on the distribution of Ecrg4 (Riken150001O15) gene expression throughout the mouse spinal cord, including other cell types, is available at http://mousespinal.brain-map.org/imageseries/show.html?id=100028769. F: Quantitative gene expression by RT-qPCR. After extracting tissues from donor adult mice (n = 12), highest *Ecrg4 *expression was found in the dissected choroid plexus. Data was calculated using the ∆ΔCt method and normalized to the values obtained in testes. Similar findings were obtained in a survey of male rats (not shown) and low gene expression was detectable in all tissues. Error bars represent mean ± standard deviation, n = 5.

The fact that the *Ecrg4 *gene is down-regulated by hyper-methylation in many cancers suggests that its epigenetic control plays a role in the transformation of normal cells to cancer [12-18]. Yet little is known regarding its pathological, let alone, physiological, function in the CNS except that it is implicated in senescence [19], suggesting that it may play important physiological functions in the brain. It was therefore particularly interesting to note that publicly available genomic data bases including Genepaint [11], the St. Jude Brain Gene Expression Map (BGEM) [20], and the Allen Brain Atlas [21], showed that *Ecrg4 *gene expression was significantly higher in the CP than other CNS tissues in both the developing and adult mouse CP (Figure 2). With this in mind, we explored (1) the relative gene expression levels of *Ecrg4 *compared to other tissues, (2) whether augurin, the 14 kDa peptide product of *Ecrg4*, is expressed and secreted by the CP and (3) how augurin expression is linked to CNS development and (4) in the CNS injury response.

We show here that there is elevated expression of the *Ecrg4 *gene in the CP compared to other CNS and peripheral tissues and that its product, augurin plays a role in CNS injury. In the adult, *Ecrg4 *expression in the CP is decreased following injury, but over-expression induced by gene transduction is growth inhibitory in the subependymal zone. In development, gene knockdown with antisense RNA is growth stimulatory. Taken together, we suggest a novel homeostatic role for augurin in the CNS and propose that a dysinhibition model would explain how augurin regulates cell destiny at the ependymal-subependymal interface after CNS injury. If correct, the canonic epigenetic methylation of the *Ecrg4 *gene that characterizes augurin production [17] may forecast the progenitor response to injury, and may therefore implicate the CP in defining the regenerative capacity of the CNS.

## Materials and methods

### Animals, tissues and processing

All animal studies were performed with approval of, and strict adherence to, either the Institutional Animal Care and Use Committee of the University of California, San Diego (La Jolla, CA) or the Home Office (UK) depending on whether the studies were performed at UCSD or the University of Birmingham. Human tissues were collected in accordance to protocols approved by the Brown University Institutional Review Board for human studies and the Brown University Brain Tissue Resource Center (Providence, RI). The human samples evaluated here were obtained during routine autopsy from a 61 year-old male with no history of neurological disease. Tissue expression studies were performed using tissue harvested from 20-25 g Balb C mice that were collected after either cervical dislocation or decapitation, immediately frozen on dry ice and kept at -80°C until analyzed. Male Sprague Dawley rats (n = 3, 200-250 g) were killed with CO_2 _inhalation and immediately perfused with 4% formaldehyde in phosphate-buffered saline (PBS), pH 7.4. Breeding zebrafish (*Danio rerio*) were maintained at 28°C on 14 h light/10 h dark cycle and fertilized eggs for morpholinos injections were retrieved from natural spawning and used immediately.

### Antibodies

A polyclonal antibody was raised in chickens by immunization against a recombinant fusion protein that included the amino acid sequence 71-148 of the full length predicted product of the *Ecrg4 *open reading frame (ORF) by commercial contract to GenWay Biotech, Inc., (San Diego, CA, USA) and antigen affinity purified to generate purified IgY. Pre-immune IgY was obtained from the same chicken prior to immunization, immunoglobulin purified and used as a control. Horseradish peroxidase (HRP)-conjugated goat anti-chicken antibody (BioRad, Hercules, CA, USA) was used as the secondary antibody in immunoblots. For immunolabeling of augurin in rat tissue, biotinylated donkey anti-chicken IgG (Stratech, Suffolk, UK) was used for immunoperoxidase detection and Alexa Fluor^® ^488 or Alexa Fluor^® ^594 (Invitrogen, Paisley, UK) conjugated goat anti-chicken IgG for immunofluorescence labeling. In human tissue, the secondary antibody used was biotinylated goat anti-chicken IgG (Vector Laboratories, Burlingame, CA, USA).

### Adenovirus (Ad)

Ad vector containing either a transgene for Ecrg4 (Ad_*Ecrg4*_) was prepared according to manufacturer's instructions using the AdEasy XL Adenoviral Vector System (Agilent Technologies, Santa Clara, CA, USA). Primers used to amplify the human *Ecrg4 *ORF were 5'-TATGTCGACCCGCCATGGCTGCC-3' (forward) and 5'-TATAAGCTTAGTAGTCATCGTA-3' (reverse). Adenovirus was produced in human embryonic kidney (HEK) 293 cells and purified and titered with the Adeno-X Virus Purification Kit (Clontech Laboratories, Inc., Madison, WI, USA). Ad vector containing a transgene for green fluorescent protein GFP *(*Ad_*GFP*_*) *used as a control for Ad infection or to visualize infected cells after intra-cerebro-ventricular (i.c.v.) injection was obtained commercially (Vector BioLabs, Philadelphia, PA, USA).

### Recombinant protein production

Human *Ecrg4 *encodes a 148 amino acid protein (Ensembl: ENSG00000119147), which contains a leader peptide at residues 1-30 and has predicted processing sites to generate peptides of 3-17 kDa (Figure 1). One processed form of the protein encoded by *Ecrg4 *has been termed augurin (residues 31-148) [22] but a single putative pro-hormone cleavage site at residues 68-71 yields two putative peptide hormones at residues 71-148 and 31-70. We have named the fragment 31-70 as ecilin after the EC part of ***Ec****rg4*, and the fragment 71-148 as argilin after the RG part of *Ec****rg****4*. This nomenclature is used throughout this report. A second predicted proteolytic consensus site for thrombin cleavage generates C-terminal ∆16 sequences when incubated with thrombin (Knowling *et al*, in preparation). Recombinant augurin derived from the human cDNA sequence and corresponding to amino acids 31-148 of the *Ecrg4 *ORF was cloned into the pET15b vector following codon and structural optimization and then over-expressed in BL21DE3p*LysS E. coli *(Promega, San Luis Obispo, CA, USA). The protein was purified to homogeneity by ion-exchange and gel permeation chromatography.

### Penetrating CNS injury model

Adult male Sprague-Dawley rats (n = 3, 200-250 g) were maintained under standard conditions with access to food and water *ad libitum*. Prior to anesthesia, rats received a subcutaneous injection of buprenorphine (0.3 mg/kg; Alstoe Animal Health, York, UK) for analgesia. Rats were then anesthetized with 5% isofluorane (Abbott Laboratories Ltd, Kent, UK) in oxygen (1.7 L/min). A simple penetrating CNS injury model used for these studies is based on a classical injury model [23-25]. Briefly, a sagittal incision was made over the dorsum of the rat head to expose the skull, and a knife lesion (3 mm depth) was made using an ophthalmic knife (Unitome knife, 3.0 mm, BD Waltham, MA, USA) in the right cerebral cortex under aseptic conditions. Rats were killed at 1 day post-lesion and tissues processed for histological analyses as described below.

### I.c.v. injection of Ad_GFP _and Ad_Ecrg4_

Following administration of anesthesia as described above, rats (n = 3 each group) were placed in a stereotactic frame and a sagittal incision was made over the dorsum of the head to expose the skull surface and identify bregma. The coordinates used for injection into the lateral ventricle were: 1 mm posterior to bregma and 1.5 mm lateral to the midline. With a micro drill, the skull was punctured and using a Hamilton syringe placed 4 mm deep into the brain, 20 μl (2 × 10^9 ^viral particles in PBS) of Ad_*Ecrg4 *_for over-expression or Ad_*GFP *_for control were injected i.c.v. After injection, the syringe was left in place for 1 min to minimize reflux. Rats were returned to housing and received a daily intraperitoneal (i.p.) injection of 5-bromo-2'-deoxyuridine (BrdU, 10 mg/200 g) on days 2-6 and brains were removed and processed for analyses as described below.

### Database mining and presentation

Gene expression profiles and *in situ *hybridization data for *Ecrg4 *(human *C2orf40 *[EMBLCDS: AAH21742]) and mouse Riken*1500015O10Rik *[EMBLCDS: AAH02254]) distribution in the CNS, were mined from data posted and publicly available at http://www.genepaint.org[11] and http://www.allenbrainatlas.org[21]. Partial representative data presented here is accordance to their citation policies [11] and readers are referred to the complete data sets describing *Ecrg4 *gene expression that are available at their respective websites for further, in depth analyses.

### Immunoblotting, ELISA and RT-quantitative PCR

#### Immunoblotting

To determine the molecular weight of the *Ecrg4 *peptide product expressed in the rodent choroid plexus, total protein was extracted from adult male rat choroid plexuses by homogenization and sonication in 1× reducing lithium dodecyl sulfate (LDS) buffer (Invitrogen). Protein was size-fractionated by polyacrylamide gel electrophoresis (PAGE) on a 4-12% bis-tris gradient gel run in 2-(N-morpholino) ethanesulfonic acid (MES) buffer and transferred to a polyvinylidene fluoride membrane. Chicken anti-augurin antibody or pre-immune IgY (GenWay) was used to detect augurin. Horseradish peroxidase (HRP) conjugated goat anti-chicken antibody (BioRad) was used as the secondary antibody in Western blots. An IVIS^® ^Lumina imaging system (Caliper Life Sciences, Hopkinton, MA, USA) was used to detect the chemiluminescent signal.

#### ELISA

Augurin protein secreted into conditioned media by primary human choroid plexus epithelial cells was quantified by competition ELISA (Peninsula Laboratories LLC, San Carlos, CA, USA) according to manufacturer's instructions.

#### Quantitative RT-PCR (RT-qPCR)

To determine relative gene expression levels in different tissues, total RNA from 18 different tissues harvested from 6 month-old male C57BL/6 mice (n = 12) was extracted using the RNeasy Mini Kit (Qiagen, Valencia, CA, USA) and reverse transcribed using the iScript cDNA Synthesis Kit (BioRad). All RT-qPCR runs were performed in duplicate, and the amplification cycle threshold (Ct) for *Ecrg4 *was normalized to *glyceraldehyde-3-phosphate *(*GAPDH*) using SYBR green detection (BioRad). *Ecrg4 *expression levels were normalized to levels in testes. Amplification cycle parameters were as follows: 95°C denaturation, 60°C annealing and 72°C elongation. Efficiencies for both primer sets as determined by standard curve analysis were 95-100%. Primer sequences for *Ecrg4 *were as follows: *Ecrg4 *forward 5'-AAGCGTGCCAAACGACAGCTGTGGGAC-3'; *Ecrg4 *reverse 5'-TTAATAGTCATCATAGTTGACACTGGC-3'; GAPDH forward 5'-GCACAGTCAAGGCCGAGAAT-3'; GAPDH reverse 5'-GCCTTCTCCATGGTGGTGAA-3'.

### Immunostaining

#### Human paraffin-embedded brain tissue

Human CP tissue was obtained at autopsy, fixed in neutral-buffered formalin (NBF), and paraffin embedded. After preparing 8 μm sections, tissue was deparaffinized and rehydrated. For antigen retrieval, the tissue was incubated in 10 mM citrate, pH 6 at 85°C for 20 min and rinsed. Sections were quenched with peroxidase blocking reagent (Dako, Carpinteria, CA, USA) and blocked with 5% normal goat serum and incubated in 0.5 μg/ml chicken anti-augurin IgY (GenWay). Biotinylated goat anti-chicken secondary antibody (Vector Laboratories) was used at 2 μg/ml. Augurin immunoreactivity was detected using the standard avidin-biotin immunoperoxidase complex method as described elsewhere [26] with reagents from Vector Laboratories.

#### Rat brain tissue analyses

Sham and injured rats were euthanized in a CO_2 _chamber, trans-cardially perfused with PBS and then fixed with 4% paraformaldehyde (PFA) in PBS pH 7.4. Brains were dissected and post-fixed overnight at 4°C in PBS containing 20% sucrose and 4% PFA. Tissues were then rinsed overnight at 4°C in PBS containing 30% sucrose and rapidly frozen into OCT compound (Miles Laboratories, Naperville, Il. USA) on dry ice and stored at -80°C. For the analyses of the injury response, 15 μm thick coronal brain sections were placed on charged microscope slides and stored at -20°C. For analyses of the effects of Ad_*Ecrg4 *_transgene over-expression, frozen coronal brain sections (28 μm thick) were collected and stored free-floating in cryoprotectant solution (50% 0.05 M sodium phosphate buffer, 30% ethylene glycol, Sigma, Dorset, UK., 20% glycerol, Sigma). To block non-specific binding, tissue sections (15 μm) were first incubated for 20 min at 25°C in PBS containing 0.3% Tween 20, 2% Bovine Serum Albumin (BSA, Jackson ImmunoResearch, Suffolk, UK) and 15% normal goat serum (Vector, Peterborough, UK). Sections were drained and incubated at 4°C overnight in chicken anti-augurin IgY (2 μg/ml). Sections were then rinsed in PBS containing 0.3% Tween and then incubated in 1 μg/ml 488 Alexa Fluor^® ^goat anti-chicken (Invitrogen) for 45 min at 25°C. Finally sections were rinsed and mounted with mounting media containing 4',6-diamidino-2-phenylindole for nuclear counterstain (DAPI, Vector Labs). Staining was visualized under an epifluorescent (Zeiss) or confocal microscope (Zeiss LSM510). Pre-immune IgY was used as negative control for augurin staining. In some instances localization of augurin was performed by immunoperoxidase staining, using the standard avidin-biotin peroxidase complex method (Vector) and reaction visualized with diaminobenzidine (DAB, Vector) as substrate.

Free-floating sections were also used for immunostaining of augurin, GFP, nestin and BrdU after i.c.v. injections of Ad_*GFP *_and Ad_*Ecrg4*_. and after i.p. injections of BrdU. For BrdU staining, sections were initially treated with 1 M HCl at 45°C for 30 min prior to immunostaining. To block non-specific binding, sections were incubated with 2% BSA and 15% normal goat serum in PBS pH 7.4 for 1 h. Sections were then incubated with either chicken anti-augurin IgY (0.5 mg/ml) for 1 h at room temperature (RT) or with sheep anti-BrdU IgG (10 mg/ml, Sigma) overnight at 4°C. Finally the sections were rinsed and incubated with 1 mg/ml of either Alexa Fluor^® ^594 goat anti chicken or donkey anti-sheep (Invitrogen) for 45 min at 25°C. Sections were then mounted on slides, covered with mounting media containing DAPI and the staining visualized under an epifluorescent (Zeiss) or confocal microscope (Zeiss LSM510). Mouse anti nestin was obtained from BD Biosciences (556309), blocked in 10% normal goat serum in PBS/Tween/2%BSA and incubated at 1/200 in PBS/Tween/BSA overnight at 4°C. Alexa 594-labeled anti mouse 1/1000 was incubated for 45 min at RT for fluorescence labeling (Invitrogen). Pre-immune IgY was used as negative control for augurin staining. Multiple Z-stacks were taken from each animal of the BrdU staining observed in the SVZ and BrdU-positive cells were counted manually while blinded to the treatment group of the animals. GFP was detected by direct fluorescence. To determine the significance of differences in BrdU-positive cells in Ad_*GFP *_- and Ad_*Ecrg4 *_- injected animals, statistical analysis was performed using Student's T-test with equal variances after confirming independence of the variables.

#### In situ Hybridization

Restriction enzyme linearized (Sal I) plasmid pCMV-Sport 6 expressing the mouse *Ecrg4 *cDNA was purchased from Origene (#MC200116) and used as template for the generation of digoxigenin-labeled RNA probe using T7 polymerase as indicated by manufacturer's recommendations (# 11-175-025-910, Roche, Indianapolis, IN, USA). Control RNA probes were generated from pSPT18-Neo plasmid while labeling efficiency was verified by dot blot comparison of RNA probes with pre-labeled standards provided in the kit. Rat tissues were processed as described for immunohistochemical analyses. Brain tissue sections (15 μm) were washed in PBS, permeabilized with PBS containing 0.1% Triton X-100 (Sigma) and acetylated for 15 min in 0.1 M triethanolamine (Sigma) pH 8.0 containing 0.25% (v/v) acetic anhydride (Sigma). Sections were then rinsed in 0.2 × SSC (Sigma) and incubated for 15 min at 52°C in pre-hybridization buffer (4 × SSC containing 50% de-ionized formamide, Sigma). Sections were hybridized for 16 h at 52°C with DIG-labeled complementary RNA or control RNA probe diluted in hybridization buffer containing 40% de-ionized formamide, 10% dextran sulfate (QBiogene, Cambridge, UK) 1× Denhardt's solution (Sigma), 4 × SSC, 10 mM DTT (Roche Diagnostics, Mannheim, Germany), 1 mg/ml yeast t-RNA (Invitrogen) and 1 mg/ml denatured and sheared salmon sperm DNA (Roche Diagnostics). Non-specific binding was removed by washes in 2 × SSC at 37°C and single stranded RNA probe digested by incubating the sections for 30 min at 37°C in NTE (500 mM NaCl, 1 mM EDTA, 10 mM Tris pH 8.0) buffer containing 20 μg/ml RNase A (Worthington Biochemical, Reading, UK). Sections were then rinsed in 0.1 × SSC at 45°C for 1 h, rinsed in 100 mM Tris pH 7.5 containing 150 mM NaCl. Sections were then blocked with 100 mM Tris buffer containing 0.1% Triton X100 and 10% normal sheep serum (Jackson ImmunoResearch) for 30 min, drained and incubated for 2 h at 25°C in anti-digoxigenin-POD, Fab fragment (1/50 Roche Diagnostics) diluted in 100 mM Tris, 0.1% Triton X100 and 2% normal sheep serum. Sections were rinsed, incubated in DAB substrate (Vector) for 10 min, rinsed in distilled water, dehydrated and mounted. Slides were analyzed and pictures taken under a bright field microscopy (Axioplan, Zeiss).

#### Cell culture and transduction of human choroid plexus epithelial cells

Primary human CPe cells were obtained from ScienCell Research Laboratories (Carlsbad, CA, USA) and cultured according to distributor's recommendations. HEK 293 (ATCC, Manassas, VA, USA) cells were cultured under standard conditions as described previously [27]. CPe and HEK cells were transduced with Ad_*Ecrg4 *_or Ad_*GFP *_at a multiplicity of infection (MOI) of 100. Expression of the transgenes was determined by RT-PCR and immunoblotting as described above.

#### Zebrafish morpholino treatments and analyses

A 25 base pair anti-sense morpholino, synthesized by GeneTool, was designed to target the 5' untranslated region to block protein translation of zebrafish *Ecrg4a *(zgc:112443 [EMBLCDS:AAH93311]). A standard control morpholino was also purchased to assess non-specific effects caused by injection and morpholino dosing. Specifically, the sequence of the standard negative control morpholino used in the experiments described here was: 5'-CCTCTTACCTCAGTTACAATTTATA-3' which targets a human β-globin intron mutation and has not been reported to have other targets or generate any phenotypes in any known Zebrafish test systems. The *Ecrg4a *morpholino (5'-TTCTGCTCTTCTCCTTCTTCTCTGT-3') targets the 5' untranslated region and recommended by morpholino selection algorithms accessed through http://www.zfin.org.

The *Ecrg4a *open reading frame was cloned using PCR from the zgc:112443 cDNA clone (Open Biosystem) and the fragment ligated into a pCS2+ expression vector. Capped mRNAs were synthesized with linearized *Ecrg4a*-pCS2+ by mMessage SP6 kit and purified with a Qiagen RNeasy mini Kit. Microinjections into 1-2 cell embryos used 1 nl of 0.5 mM control morpholinos, or 1 nl of 0.06 to 0.125 mM anti-*Ecrg4a *morpholino for low or high dosages respectively in a Warner Instrument Picoliter Pressure Line injector (PLI-100). In mRNA rescue experiments, 1nl solution containing 25 pg of capped *Ecrg4a *mRNA was injected with 0.125 mM *Ecrg4*a morpholino. Photographs were taken at different stages and phenotypic changes documented. To visualize the CNS effects of anti-*Ecrg4a *morpholino injections, the area of CNS edema was identified by lasso and cropped from images, printed and cut from photographs that had been equally enlarged using Preview (Apple, Cupertino, CA, USA). These areas were measured and reported as artificial optical units. Quantification of the effect of *Ecrg4 *and control morpholinos on the size of the zebrafish ventricles was also performed by image analysis using NIH imageJ of stage-matched 48 h embryos. Data was exported to MS-Excel for analyses.

#### Whole-mount immunocytochemistry of zebrafish embryos

embryos (48 h) were fixed in 4% PFA in PBS overnight at 4°C and permeabilized with -20°C acetone. After blocking with 15% normal donkey serum, rabbit polyclonal anti-H3P and mouse monoclonal zrf-1 (anti-GFAP) antibodies were incubated with embryos at 4°C overnight. After washing, Alexa Fluor^® ^488-labeled anti-mouse and Alexa Fluor^® ^555-labeled anti-rabbit secondary antibodies were used for detection and the embryos were subsequently mounted in custom made chambers for imaging by laser scanning confocal or epi-fluorescence microscopy.

#### Quantification of cell proliferation in zebrafish embryonic brain

In order to measure the effects of the anti-ECRG4 morpholino on cell proliferation, the z-projections of confocal stacks obtained from H3P/GFAP double staining images were converted to binary images by adjusting the threshold differentiating H3P positive cells from background. The amount of signal by was determined by measuring the percentage of area occupied by the H3P positive cells in brain regions defined by GFAP staining in projected images. The 'analyze particle' function in NIH ImageJ was used to perform the measurement and data was exported to MS-Excel for analyses.

## Results

### Expression of Ecrg4 in the mammalian choroid plexus

An alignment of the proteins encoded by the *Ecrg4 *open reading frame demonstrates the extent of sequence homology between species (Table 1 and Figure 1). Accordingly, we generated a chicken polyclonal antibody specific to the more conserved C-terminus of augurin for use in immunohistochemistry and ELISA assays, reasoning that the antibodies generated would cross-react among different species. Whereas the *Ecrg4 *open reading frame encodes a 148 amino acid protein however, post translational processing can potentially produce 8 peptides (Figure 1B) that include the (1) the full length Ecrg4 protein, (2) cleavage of the leader peptide from *ECRG4 *to generate the fragment called augurin [10], and two others that we termed ecilin (EC) and argilin (RG) that are predicted to be generated by furin-like cleavage and another two forms of augurin and argilin that are predicted from a thrombin consensus cleavage site that would release the last 16 amino acids of the carboxyl terminus peptide (Figure 1) to generate C∆16-augurin and C∆16-argilin. For purposes of clarity, this nomenclature is used throughout this manuscript.

**Table 1:** The *ECRG4 *open reading frame shows high evolutionary conservation

Species	Gene	Protein	Homology to human
*Homo sapiens*	C2orf40	NP_115787.1	

*Mus musculus*	1500015O10Rik	NP_077245.1	84%

*Rattus norvegicus*	RGD1305645	XP_343563.1	83%

*Pan troglodytes*	LOC459473	XP_001164223.1	99%

*Bos taurus*	*ECRG4*	NP_001033202.1	81%

*Canis lupus familiaris*	LOC611190	XP_853925.1	89%

*Gallus gallus*	LOC771055	XP_001234361.1	58%

*Danio rerio (A)*	zgc:112443	NP_001017697	47%

*Danio rerio (B)*	BC095747.1	P0CAX4.1	42%

Analyses using the open-access resources Gene Paint [11] and the Allen Brain Atlas [21] revealed that the expression of *Ecrg4 *in the developing CNS of the mouse embryo at stage E14.5 localizes primarily to the choroid plexus (Figure 2A). While there are also other loci of abundant probe hybridization indicating *Ecrg4 *gene expression, for example in heart and cartilage [10,28], the localization pattern in mouse CNS may suggest a role in CP and ependymal function. In the developing mouse embryo, the localization of *Ecrg4 *mRNA in CPe is clearly evident (Figure 2B) and is also present in adult brain (Figure 2C). A less intense signal was also detected throughout the brain parenchyma in what appear to be neurons by immunohistochemistry of human and rat brains (Miller *et al*, and Gonzalez *et al*, in preparation). In the adult mouse brain, *Ecrg4 *mRNA localized to CPe (Figure 2D) and the epithelial lining of the central canal (Figure 2E, inset). Probe hybridization was also observed in the gray matter of the spinal cord (Figure 2E). A survey of various tissues by real-time PCR underscored the fact that the CP is a major site of *Ecrg4 *gene expression in the adult mouse (Figure 2F) and similar results were found in rat (data not shown). Although gene expression is detectable in other tissues (i.e. adrenal, pancreas, heart and skin), expression was relatively low compared to the signal from CP tissues.

### Localization of the Ecrg4 product augurin to the CPe

**Figure 3:**
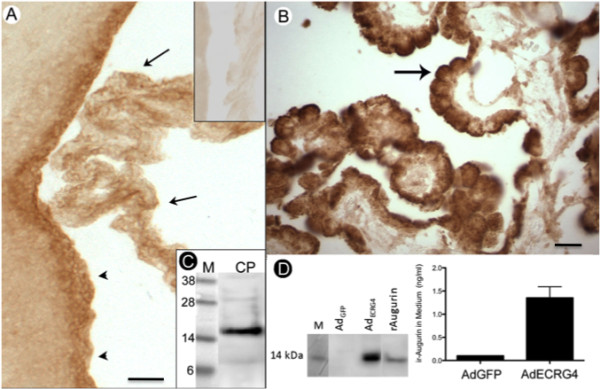
**The choroid plexus expresses augurin, a 14 kDa processed protein form of the *ECRG4 *ORF corresponding to amino acids 31-148**. A: Immunostaining in rat brain: Immunoreactive augurin was present in the choroid plexus (arrow) and ependyma (arrowhead) of the third ventricle in the adult rat compared to pre-immune IgY control (top inset). Scale bar = 20 μm B: Immunostaining in human brain: Adult human tissue showed that similarly, augurin staining localized primarily to the epithelial cell layer of the human CP (black arrow). Scale bar = 20 μmC: Immunoblotting of rat choroid plexus. When rat CP tissue was extracted and analyzed by immunoblotting as described in the text, an immunoreactive 14 kDa band was detected. It corresponds to the anticipated molecular weight of augurin. D: Augurin secretion *in vitro*: When adult primary human CPe cells were transduced with an adenovirus vector containing the green fluorescent protein gene (Ad_GFP_), they were fluorescent (not shown) and like untreated CP cells in culture, do not express augurin. When these cells are transduced with adenovirus vector containing *Ecrg4 *cDNA (Ad_*Ecrg4*_), augurin protein can be detected in RIPA lysates by immunoblotting and the Ad_*ECRG4*_-transduced CPe cells secrete augurin into the conditioned medium that can be measured by ELISA but not detected by immunoblotting. M = molecular weight markers and rAugurin = recombinant augurin used as standard and purified from, *E coli*.

Augurin-immunoreactive protein localized to CP and ventricular ependymal cells of adult rat brain (Figure 3A) as predicted by the gene expression data shown in Figures 2C, D and 2F. There was no specific staining observed in tissue incubated with the pre-immune IgY (Figure 3A, inset) and a similar pattern was detected in mouse CNS (not shown). In human choroid plexus (Figure 3B), cytoplasmic and in some cells, apical staining was observed (Figure 3B, arrow) that appeared in many instances to be localized to the plasma membrane suggesting polarized cell surface localization. Immunoblotting with the anti-augurin antibody after PAGE size fractionation of rat CP extracts, showed that the endogenous form of CP-derived immunoreactive protein is14 kDa (Figure 3C), the predicted molecular weight of augurin *Ecrg4*(31-148). This size is consistent with processing to remove the leader sequence *Ecrg4*(1-30) as predicted by Mirabeau *et al *[20]. Unlike neurohypophyseal extracts (A. Roberton, personal communication and [20]), immunoblotting did not reveal significant presence of the further processed peptides (Figure 1) [10] including ecilin, the peptide corresponding to *Ecrg4 *amino acids 31-70, or argilin, the peptide corresponding to the C-terminus *Ecrg4 *amino acids 71-148 (Figure 1). Longer detection times of the immunoblotting membranes show trace amounts of an 8 kDa peptide, that could correspond to argilin and a 28 kDa peptide could be an augurin dimer that is not dissociated by sample boiling or detergent disruption (S.E. Knowling, unpublished observation).

### Augurin was secreted into culture media conditioned by transduced human CPe cells

The processed product of the *Ecrg4 *gene is presumed to be a secreted protein [19,28] because the ORF contains a consensus leader sequence that directs augurin into the endoplasmic reticulum and Golgi apparatus for translocation outside of the cell. To determine whether CP-derived augurin could be secreted in to media by CPe cells, we used a quantitative ELISA assay to analyze the conditioned media of Ad_*Ecrg4*_-transduced cultured primary human CPe cells versus control cells (Ad_GFP_-transduced CPe cells). Presumably because Ecrg4 is down regulated in injury phenotypes like cell culture (unpublished observations), we were not able to detect endogenous *Ecrg4 *protein expression in this or several other cell lines assayed (Figure 3D and unpublished results). It is possible that *Ecrg4 *expression is lost as cells are selected for their ability to grow in culture because Ecrg4 expression has been linked to senescence and cell cycle arrest [12-18]. Following Ad_*Ecrg4 *_gene transduction and consequent transgene over-expression however, the CPe cells do express, process and secrete augurin (Figure 3D). CPe cells transduced with Ad_*Ecrg4 *_expressed an immunoreactive augurin that could be detected by immunoblotting of cell lysates and ELISA of conditioned media (Figures 3D, E). As expected, no signal was observed in Ad_*GFP*_-transduced CPe cells or conditioned media. Together these results suggest that there is cell-type processing to produce a 14 kDa augurin and when expressed by CPe cells, it can be secreted into media.

### Immunoreactive augurin in the rat choroid plexus decreased following CNS injury

**Figure 4:**
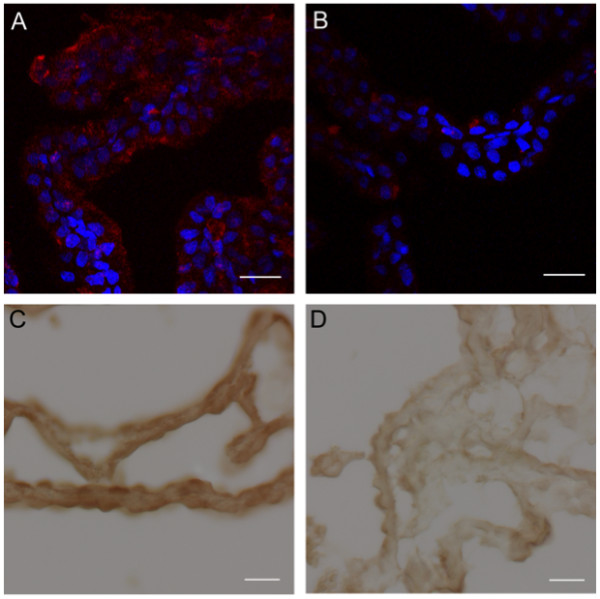
**Augurin immunoreactivity and *Ecrg4 *gene expression decreases in the CP following CNS injury**. A and B: Augurin immunoreactivity decreased in rat choroid plexus after penetrating CNS injury. A: Augurin immunoreactivity in the brains of control rats was, as anticipated, readily detectable in the cytoplasm of CPe cells. In certain areas, this staining appeared apical. B: There was an apparent decrease in augurin at 1 day post injury (d.p.i.). Augurin: red, DAPI: blue, scale bar = 20 μm. C and D: *Ecrg4 *gene expression decreased in rat choroid plexus after penetrating CNS injury. C: *Ecrg4 *gene expression in the brains of control rats was, as anticipated, readily detectable by *in situ *hybridization in the cytoplasm of CP epithelial cells. D: As shown in Panel D however, there was an apparent decrease in *Ecrg4 *gene expression staining in the CP-containing sections processed from animals 24 h post injury (d.p.i.). Scale bar = 20 μm.

Although *Ecrg4 *gene expression is well described as down-regulated in cancer and inversely correlated with cell proliferation [13-16], there is little known regarding its fate and distribution in the CNS [19]. To examine whether augurin might participate in the CP response to CNS injury, we first asked if there is a change in augurin immunoreactivity in CP after a penetrating injury to the cerebral cortex in the adult rat. Previous studies have established that significant changes in CP function accompany CNS injury, stroke and trauma [4,8,29,30]. As shown in Figure 4, augurin protein in rat CPe cells was decreased 24 h after penetrating CNS injury (Figure 4B relative to 4A). Furthermore, because *in situ *hybridization shows that gene expression is decreased 24 h after injury (Figure 4D relative to 4C), the loss of augurin staining is presumed to represent increased secretion and inhibited gene expression.

### Functional consequences of Ecrg4 expression after the cortical stab model of CNS injury

**Figure 5:**
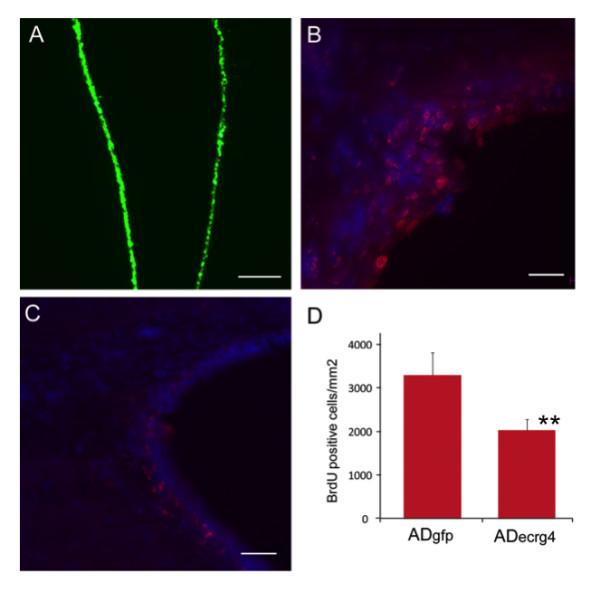
***Ecrg4 *over-expression inhibits injury-induced proliferation of subependymal progenitor cells**. A: Transduction of ependyma with adenovirus vector containing the green fluorescent protein gene (Ad_GFP_). If Ad_*GFP *_was injected i.c.v. such that target cells were labeled prior to the penetrating injury, ependymal cells throughout the ventricles were transduced as indicated by the GFP staining of ependyma (green). Scale bar = 100 μm. B: Cell proliferation after penetrating injury after Ad_GFP_. To monitor cell growth after injury, animals that had been injected with Ad_GFP_, received daily injections of BrdU i.p. as described in the text and the incorporation of BrdU into DNA was evaluated by immunohistochemistry. Brdu: red, DAPI: blue, scale bar = 20 μm. C: Cell proliferation after penetrating injury and Ad_Ecrg4_. If instead of Ad_GFP _as above, the target ependymal cells were transduced by i.c.v. injection of adenovirus containing *Ecrg4 *(Ad_*Ecrg4*_), there was a decrease in BrdU-labeled cells in the subependymal zone. Scale bar = 20 μm. D: Quantification of cell proliferation after penetrating injury after Ad_GFP _or Ad_Ecrg4. _When apparent differences in proliferating cell number were quantified, a significant difference in proliferating cell number was observed (*p *< 0.01). Error bars represent mean ± standard deviation (n = 4).

To ask whether there might be a correlation between augurin expression in the CPe and recovery from injury, we examined the effect of *Ecrg4 *transgene delivery, and thus augurin over-expression, on BrdU uptake in SVZ cells following an i.c.v. injection of Ad_*Ecrg4 *_versus Ad_*GFP*_. We hypothesized that because *Ecrg4 *is down-regulated in proliferating cells like tumors and cells in culture [13-17,28] but upregulated in senescent cells [19], then augurin over-expression might affect the proliferation response to injury in the SVZ. Accordingly, we injected either Ad_*Ecrg4 *_or Ad_*GFP *_into rat brains i.c.v. to transduce ependymal and CPe cells [31]. We then injected 5-bromo-2'-deoxyuridine (BrdU) i.p. to label mitotic cells proliferating in the SVZ following CNS injury [6]. First, we confirmed the ependymal localization of transduction after i.c.v. injections of Ad by examining GFP staining (Figure 5A). These findings confirmed the adenoviral tropism for ventricular ependyma that has been used by others for gene delivery to CPe and peptide delivery to CSF [32]. In uninjured adult animals, very little BrdU incorporation has been observed in the SVZ of the brain [33]. After cortical stab injury in Ad_*GFP*_-treated rats, there were BrdU-labeled cells that were detectable by immunohistochemical staining for BrdU (Figure 5B, red). However, the number of BrdU positive cells was significantly decreased with Ad_*Ecrg4 *_injections (Figures 5C and 5D). Taken together, these data suggest that augurin produced by ependyma and CPe, could have a role in regulating the proliferation rate of subependymal cells presumably, NSPCs. If so, it was important to test the possibility that the loss of augurin immunoreactivity that is observed after injury (see Figures 4A and 4B) might dysinhibit neuroepithelial progenitors, thereby allowing them to proliferate.

### Ecrg4 targets nestin-positive neuroepithelial progenitor cells

**Figure 6:**
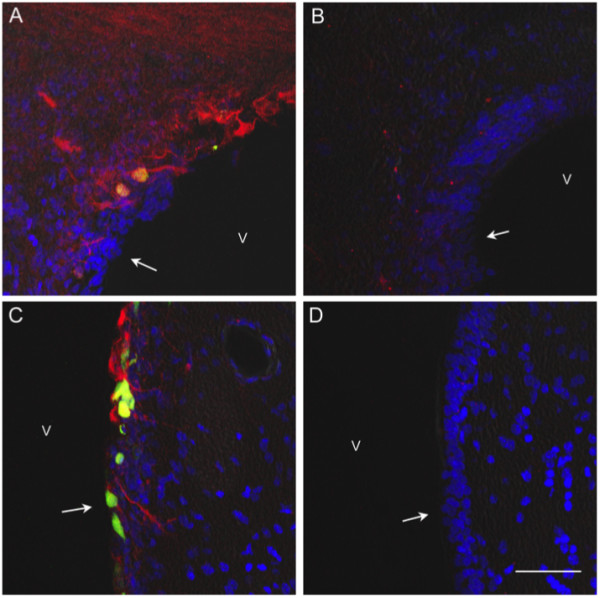
**Nestin immunoreactivity is decreased in the subependymal zone following *Ecrg4 *over-expression**. A and C: Nestin immunoreactivity in rat subependymal zone. When examined under low (Panel A, 200×) or higher (Panel C, 400×) magnification, nestin immunoreactivity labeled neuroepithelial progenitor cells that were present and proliferating after the i.c.v. injection of Ad_*GFP *_into injured rat brains. Nestin was also detectable in the ependymal cell layer as denoted by arrows in the ventricle (v) where it co-localized with GFP positive cells. Blue = DAPI, Red = nestin, green and yellow = GFP in Ad_*GFP *_positive cells. B and D: Nestin immunoreactivity decreased in subependyma after AD_Ecrg4 _injection. There was decreased nestin staining that was readily observed in the subventricular zone of Ad_*Ecrg4 *_treated rat brains when examined under low (Panel B, 200×) or higher (Panel D, 400×) magnification. These are the same zones where BrdU incorporation was also decreased (see Figure 5). The ependymal cell layer is denoted by arrows in ventricle (v). Blue = DAPI, Red = nestin Scale bar = 20 μm.

The possibility that *Ecrg4 *over-expression inhibits NSPC activation in the SVZ after injury, is consistent with the predicted inhibitory effects of *Ecrg4 *gene expression on other cell types, particularly tumor cells [12-18]. For this reason, it was important to determine if BrdU-labeled cells increased by injury and blocked by *Ecrg4 *over-expression, were indeed NSPCs. We therefore examined the distribution of nestin immunoreactivity in the SVZ. Nestin is expressed in dividing cells during the early stages of development and its expression levels decrease with differentiation, but levels are elevated after CNS injury [34]. As shown in Figure 6, nestin was present in the SVZ (Panel 6A, red) and in ventricular subependymal cells (Panel 6C) of Ad_*GFP*_-treated rat brains. The Ad_*Ecrg4 *_-treated rat brains however were virtually devoid of nestin staining in both SVZ (Panel 6B) ependyma and subependyma (Panel 6D). Furthermore, nestin staining in other unrelated CNS areas (e.g. subfornical organ), was unchanged by Ad_*Ecrg4 *_(not shown) indicating that the injury response affected by *Ecrg4 *over-expression is local.

### Knockdown Ecrg4 gene expression during development inhibits cell growth and induced hydrocephalus-like edema

**Figure 7:**
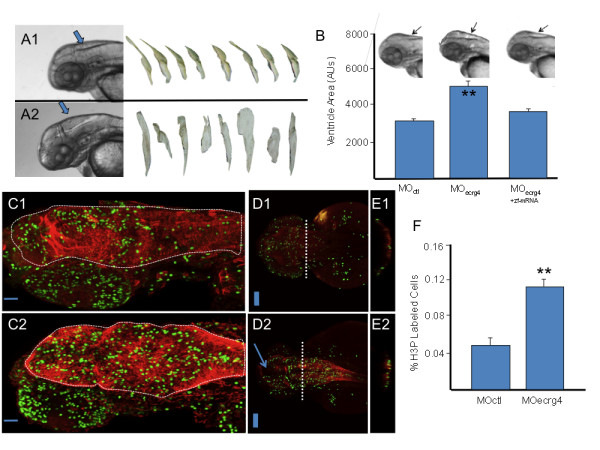
***ECRG4 *gene knockdown causes enlarged hindbrain ventricles and a hyper proliferative response in the developing zebrafish**. A: Development of Zebrafish after morpholino injection 48 hours post fertilization. In control (A1) and *Ecrg4 *(A2) morpholino treatments, the head, eyes, heart, yolk and hind ventricle (arrows) were photographed for analysis. The anti-sense *Ecrg4*a morpholino caused readily detectable hindbrain ventriculomegaly resembling a hydrocephalus-edema-like response in the CNS. The ventricles can be isolated from images for analyses (n = 8). B: Quantification of developmental effects of morpholino injection. Hindbrain ventricle size was quantified by pixel area of microscope images from n = 8 control (MO ctl), n = 8 *Ecrg4*a MO-treated embryos or (n = 8) *Ecrg4*a MO co-injected with the Zebrafish mRNA ortholog A to neutralize specific inhibition (error bars: mean ± standard deviation). C-E: Cell growth in Zebrafish 24 hours after control or Ecrg4a morpholino injection 24 hours post fertilization. Proliferating cells in control MO (top panels) from sagittal (C1), dorsal (D1) or after cross sections at dashed line (E1) can be compared to similar sections of *Ecrg4a *MO (bottom panel) injected animals by staining for phosphorylated Histone 3 (H3P, green), a marker of cell growth. H3P-positive proliferating cells are compared to that of cytoplasmic glial fibrillary acidic protein (red). The arrow (D2) highlights space corresponding to enlarged ventricles in MO-*Ecrg4 *treated embryos. The H3P positive cells are on the ventricle surface and there are more H3P cells and thicker GFAP staining in *Ecrg4a *MO. Scale bar is 100 μm F Quantification of cell growth in Zebrafish after control or Ecrg4a morpholino injection. The amount of H3P-postive cell staining was determined as described in the text and compared between treatment groups (n = 7, mean ± SEM). There were significantly more labeled cells in the Ecrg4a morpholino group. Scale bar is 100 μm.

Because *Ecrg4 *over-expression during injury results in inhibition of NSPC proliferation (Figures 4 and 5), we predicted that *Ecrg4 *gene knockdown might result in increased cell growth. Accordingly, we employed a developmental knockdown model of *Ecrg4 *gene expression *in vivo *to further analyze augurin function in the CNS. The developing zebrafish was selected while awaiting the generation of a knockout mouse model [22,35,36]. In developing zebrafish, the functional knockdown of specific genes has been proven effective [37] and it is widely accepted as a surrogate technique to mammalian gene analysis [38,39]. The model exploits transparent embryos and, because the ventricles are highly visible, and the CP well-developed in early development [40], it is well suited to test *Ecrg4 *function in the CNS. To this end, *Ecrg4*a morpholinos (MOs) were injected into developing zebrafish embryos at 1-2 cell stages using either a control MO (Figure 7A1) or an antisense *Ecrg4*a MOs (Figure 7A2). The development of embryos was then observed for 120 h. Within the first 48 h, severe defects in CNS development were observed in the *Ecrg4a *MO group including a brain ventricular hydrocephalus-like edema phenotype. A second *Ecrg4*a MO was used to confirm and validate the reproducibility of the *Ecrg4 *knockdown phenotype (not shown). Specificity of the effects of *Ecrg4*a knockdown was confirmed by near-complete rescue by quenching the morpholinos with a co-injection of the *Ecrg4*a MO with *Ecrg4*a mRNA (Figure 7B). To determine the effect of *Ecrg4*a knockdown on CNS cell proliferation, control and *Ecrg4*a MO-injected embryos were processed for whole mount immunostaining and analyzed using an antibody against phosphorylated histone-3 antibody (H3P) that is a marker for proliferating cells in developing Zebrafish [41]. In these experiments (Figures 7C, D and 7E), we observed that *Ecrg4a *MO treatment increased cell proliferation in the developing CNS. Quantitative analyses confirmed that there was increased H3P immunoreactivity in embryos when *Ecrg4 *gene expression was knocked down (Figure 7F). Cross-sectional analyses (Figures 7E1 and 7E2) suggested that the distribution of these proliferating cells is at the ventricular surface, and co-localizes with increased glial fibrillary acidic protein (GFAP) immunoreactivity. Taken together, these data suggest that *Ecrg4 *gene knockdown dysinhibits cell proliferation of GFAP-positive cells. This finding is consistent with (1) the inhibitory response observed with *Ecrg4 *over-expression in rat brain (Figure 5D) and (2) the proliferative response of endogenous NSPCs after injury (Figure 6D) when augurin staining transiently disappears (Figures 4A and 4B). We can therefore speculate that endogenous augurin might regulate CNS cell proliferation, injury response and, because its targets are progenitors, indirectly control regeneration after CNS injury.

## Discussion

In this study, we examined whether there are physiological and pathophysiological consequences to the expression, over-expression and knockdown of the *Ecrg4 *gene in the CNS. The results across the four species evaluated (zebrafish, mouse, rat and human) are all consistent with the hypothesis that augurin plays a physiological role in cell growth, development and the injury response.

Immunohistochemical labeling of augurin and *in situ *hybridization of *Ecrg4 *in mouse, rat and human brain tissue sections (Figures 2, 3 and 4) showed that protein and gene expression localize in large part, albeit not exclusively, to CP and ependymal epithelial cells. Endogenous *Ecrg4 *gene expression decreased in a cortical stab model of CNS injury (Figure 4) and exogenous over-expression via an injection of Ad_*Ecrg4 *_vector i.c.v. inhibited subependymal cell proliferation (Figure 5) that correlated with decreased immunoreactivivity of nestin, a marker of NSPCs (Figure 6). Finally, *Ecrg4 *gene knockdown using RNA interference targeting *Ecrg4*a in zebrafish embryos led to a hydrocephalus-like phenotype and increased GFAP-positive cell proliferation (Figure 7). These observations are all consistent with the hypothesis that augurin can affect cell destiny in the CNS by controlling cell proliferation of NSPCs: its presence is inhibitory and its absence, dysinhibitory.

The transient loss of augurin staining after CNS injury that we observed (Figure 4B) occurred at a time of subependymal progenitor cell proliferation [34]. Because the injection of Ad_*Ecrg4 *_prevented this injury-induced loss of augurin and decreased proliferation of NSPCs, we suggest that there is a physiological link between the production of augurin in the CPe and ependyma and progenitor cells in the subependymal zone. The observation that *Ecrg4 *over-expression decreased nestin-positive cell staining supports this hypothesis. Accordingly, all of these observations are consistent with the hypothesis that one biological activity of augurin is to control SVZ cell response to injury.

The major peptide product of *Ecrg4 *gene expression in the CP appears to be the 14 kDa peptide called augurin. The immunoreactive band in mouse CP extracts resolved at approximately this molecular weight and the form produced by Ad_*Ecrg4 *_-transduced primary CPe cells co-migrates with recombinant Ecrg4(31-148) produced in *E. coli *(Figure 3C). The detection of 8 kDa and 28 kDa immunoreactive peptides that correspond to argilin (Ecrg4(71-148)) and an augurin dimer were also noted however.

We initially hypothesized that *Ecrg4*-derived peptides might be involved in regulation of CSF formation, composition and hydrodynamics because CPe cells also synthesize, secrete and respond to fluid homeostasis peptide hormones including vasopressin, natriuretic factors, growth factors and endothelin-1 [42,43]. Bioinformatic predictions also raised the possibility that the *Ecrg4 *gene can encode several secreted neuropeptide-like hormones in addition to augurin (see Figure 1) and we hypothesized that each might have disparate and distinct biological activities. For example, we observed that unlike CP, extracts of hypothalamus and neurohypophysis contain an 8 kDa argilin rather than the 14 kDa augurin [44]. Furthermore, argilin co-localized with fluid homeostasis factors including arginine vasopressin, oxytocin and atrial natriuretic peptide in the neurohypophysis, supraoptic (SON) and paraventricular nuclei (PVN) [45]. Tadross *et al *[46] demonstrated that this C-terminal fragment of augurin [*Ecrg4*(71-148)] is a secretagogue of corticotrophin-releasing hormone after i.c.v. injection. The hydrocephalus-like phenotype observed in developing zebrafish after gene knockdown could therefore still support the hypothesis that one or more of the peptide products of *Ecrg4 *are involved in regulation of fluid balance. In light of the effects of *Ecrg4 *expression on cell proliferation (BrdU) and differentiation (nestin), it is more likely that this hydrocephalus phenotype is caused by increased ventricular cell proliferation resulting in obstruction of CSF flow and drainage during development. Experiments designed to further understand how *Ecrg4 *peptide products might differentially affect fluid balance and/or cell growth and differentiation are underway.

We presume augurin to be secreted by CPe and ependyma *in vivo *because the ELISA data presented in Figure 3 show secretion by transduced primary CPe into conditioned medium. Interestingly, other cultured cells assessed in our laboratory do not secrete detectable amounts of immunoreactive augurin into culture media. Instead, neutravidin precipitation of biotin-labeled cell surface proteins identifies a 14 kDa augurin that is secreted, but retained, on the cell surface (unpublished observations). Thus it is possible that augurin, like ephrins, jagged and notch [47,48], has certain activities that are related to its anchoring onto the plasma membrane and cell-cell presentation. If so, even the 14 kDa augurin may play multifunctional roles in the CPe and ependyma depending on its bioavailability. To this end, an evaluation of the patent literature reveals that the *Ecrg4 *gene is one of very few genes identified in a differential analyses of feeder/non-feeder cell layers for their capacity to support [49] the survival of progenitor cells.

Although several previous studies have now examined *Ecrg4 *gene expression in cancer, bone development, and more recently CNS aging, the studies described here provide the first functional *in vivo *demonstration that *Ecrg4 *expression is (1) necessary for normal CNS development, (2) involved in the CNS injury response in mammals and (3) regulates cell fate in the sub-ependymal zone. In tumor cell models and in specimens derived from human biopsies, *Ecrg4 *gene expression has been related to the production of a putative secreted tumor suppressor. Decreased *Ecrg4 *gene expression is correlated with the transition from benign to malignant tumor cell growth and the degree of *Ecrg4 *gene suppression corresponds to aggressive growth [14]. The data presented here suggest the *Ecrg4 *gene expression is also involved in normal cell growth control and homeostasis. The constitutive production of augurin by CPe and ependymal cells presumably maintains a baseline growth inhibitory environment that maintains and reduces resting cell proliferation rates. With this in mind, any decreased *Ecrg4 *expression would create a local "dysinhibitory niche" that would enable cell responsiveness to other growth stimulatory factors.

Finally, it is important to note that there is a statistically significant correlation between the frequency of cytosine-phosphate-guanine island methylation in the 5' upstream non-coding region of the *Ecrg4 *gene and the degree of malignancy in several types of cancers, including choroid plexus papillomas and carcinoma [16,18,50]. As *Ecrg4 *gene expression is thought to be epigenetically regulated in cancer by hyper-methylation [13-15,17,18], it is particularly interesting to speculate that, in addition to mechanisms like transcription factors that control the response to injury, epigenetic dosing of the *Ecrg4 *gene expression by methylation might serve to regulate augurin production under physiological circumstances. If so, the data presented here predict that these epigenetic changes would dose subependymal progenitor cell responsiveness after CNS injury. Accordingly, epigenetically-driven dosing of *Ecrg4 *gene expression would forecast the ability of NSPCs to proliferate and predict outcomes after CNS injury. To this end, it has not escaped our attention that augurin agonists and antagonists of the putative peptide ligand, its putative receptor and the control of its epigenetically regulated promoter could generate next generation therapeutics that alter the normal course of recovery after CNS neurodegeneration, injury repair and regeneration.

## Conclusions

We conclude that *Ecrg4 *gene expression is inversely linked to the normal injury response in the CNS and that an unusually elevated expression of the *Ecrg4 *gene in the CP implies that its product, augurin, plays a role in CP-CSF-CNS function. The facts that (1) *Ecrg4 *expression is decreased following CNS injury, (2) *Ecrg4 *over-expression is growth inhibitory in the subependyma and (3) *Ecrg4 *gene knockdown induces a hydrocephalus phenotype in development, also suggest a novel role for augurin in the CP/ependymal biology. These data are all consistent with a model whereby an injury-induced decrease in augurin dysinhibits progenitor cells at the ependymal-subependymal interface. If so, the canonic control of its promoter by DNA methylation implicates epigenetic mechanisms in neuroprogenitor fate and function in the CNS.

## List of abbreviations used

Ad: adenovirus; BrdU: 5-bromo-2'-deoxyuridine; CP: choroid plexus; CPe: choroid plexus epithelial cell layer; CSF: cerebrospinal fluid; DNA: deoxyribonucleic acid; DAPI: 4',6-diamidino-2-phenylindole; DTT: dithiothreitol; ECRG4: esophageal cancer related gene-4; ELISA: enzyme-linked immunosorbant assay; GAPDH: glyceraldehyde-3-phosphate dehydrogenase; GFAP: glial fibrillary acidic protein; GFP: green fluorescent protein; H3P: phosphohistone H3; HEK: human embryonic kidney; HRP: horseradish peroxidase; i.c.v.: intracerebroventricular; i.p.: intraperitoneal; kDa: kilo Dalton; mRNA: messenger ribonucleic acid; MO: morpholino; NSPC: neural stem progenitor cells; ORF: open reading frame; PBS: phosphate buffered saline; PCR: polymerase chain reaction; PFA: paraformaldehyde; RT-PCR: reverse transcription-polymerase chain reaction; RT-qPCR: reverse transcription-quantitative polymerase chain reaction; PAGE: polyacrylamide gel electrophoresis; SSC: saline sodium citrate buffer; SVZ: sub-ventricular zone.

## Competing interests

The authors declare that they have no competing interests.

## Authors' contributions

AMG led the UK team and had primary responsibility for the animal preclinical studies, directing and conducting the rat CNS injuries, the immunohistochemistry and in situ hybridization, contributions to experimental design and helping write the manuscript.

SP led the molecular aspects of the studies including its design, PCR and qPCR, cloning, vectors and plasmids, participating in experimental design and analysis and helping prepare the manuscript. SYL led the zebrafish studies, data analyses, figure and manuscript preparation. MCM had primary responsibility for staining of human sections, the associated QA/QC of human studies, data analyses and figure preparation. HB helped conduct rat immunohistochemistry and CNS injury studies.

WEL assisted in the rat CNS injury and rat immunohistochemistry experiments.

AR conducted initial immunohistochemistry studies in rat and mice. XD cloned, characterized and qualified the AD_*Ecrg4 *_construct used in the studies. SEK expressed and purified recombinant augurin, ecilin, argilin and C∆16 proteins. ECG conducted Zebrafish experiments. JED designed human immunohistochemistry and acquired samples and analyses. EGS supervised and selected human sample collection, experimental approach with human tissues, immunohistochemistry, in situ hybridization and data analyses. CEJ assisted in generating original hypothesis, interpreting experimental results and manuscript preparation. RC assisted in planning of animal experiments and data interpretation.

BPE assisted in design of in situ hybridization experiments and manuscript preparation.

AB generated the original hypothesis, supervised the experimental designs and data interpretation and wrote the manuscript. All authors have read and approved the final version of this manuscript.

## References

[B1] Smith DE Johanson CE Keep RF Peptide and peptide analog transport systems at the blood-CSF barrier Adv Drug Deliv Rev 2004 56 1765 1791 10.1016/j.addr.2004.07.008 15381333

[B2] Segal MB The choroid plexuses and the barriers between the blood and the cerebrospinal fluid Cell Mol Neurobiol 2000 20 183 196 10.1023/A:1007045605751 10696509 PMC11537546

[B3] Johanson CE Duncan JA Klinge PM Brinker T Stopa EG Silverberg GD Multiplicity of cerebrospinal fluid functions: New challenges in health and disease Cerebrospinal Fluid Res 2008 5 10 10.1186/1743-8454-5-10 18479516 PMC2412840

[B4] Johanson CE Palm DE Primiano MJ McMillan PN Chan P Knuckey NW Stopa EG Choroid plexus recovery after transient forebrain ischemia: role of growth factors and other repair mechanisms Cell Mol Neurobiol 2000 20 197 216 10.1023/A:1007097622590 10696510 PMC11537552

[B5] Szmydynger-Chodobska J Strazielle N Zink BJ Ghersi-Egea JF Chodobski A The role of the choroid plexus in neutrophil invasion after traumatic brain injury J Cereb Blood Flow Metab 2009 29 1503 1516 10.1038/jcbfm.2009.71 19471279 PMC2736364

[B6] Rice AC Khaldi A Harvey HB Salman NJ White F Fillmore H Bullock MR Proliferation and neuronal differentiation of mitotically active cells following traumatic brain injury Exp Neurol 2003 183 406 417 10.1016/S0014-4886(03)00241-3 14552881

[B7] Matsumoto N Taguchi A Kitayama H Watanabe Y Ohta M Yoshihara T Itokazu Y Dezawa M Suzuki Y Sugimoto H Noda M Ide C Transplantation of cultured choroid plexus epithelial cells via cerebrospinal fluid shows prominent neuroprotective effects against acute ischemic brain injury in the rat Neurosci Lett 2010 469 283 288 10.1016/j.neulet.2009.09.060 19800935

[B8] Emerich DF Skinner SJ Borlongan CV Vasconcellos AV Thanos CG The choroid plexus in the rise, fall and repair of the brain Bioessays 2005 27 262 274 10.1002/bies.20193 15714561

[B9] National Center for Biotechnology Information (NCBI) http://www.ncbi.nlm.nih.gov/guide/

[B10] Mirabeau O Perlas E Severini C Audero E Gascuel O Possenti R Birney E Rosenthal N Gross C Identification of novel peptide hormones in the human proteome by hidden Markov model screening Genome Res 2007 17 320 327 10.1101/gr.5755407 17284679 PMC1800923

[B11] Gene Paint Resources http://www.genepaint.org

[B12] Su T Liu H Lu S Cloning and identification of cDNA fragments related to human esophageal cancer Zhonghua Zhong Liu Za Zhi 1998 20 254 257 10920976

[B13] Gotze S Feldhaus V Traska T Wolter M Reifenberger G Tannapfel A Kuhnen C Martin D Muller O Sievers S ECRG4 is a candidate tumor suppressor gene frequently hypermethylated in colorectal carcinoma and glioma BMC Cancer 2009 9 447 10.1186/1471-2407-9-447 20017917 PMC2804712

[B14] Li LW Yu XY Yang Y Zhang CP Guo LP Lu SH Expression of esophageal cancer related gene 4 (ECRG4), a novel tumor suppressor gene, in esophageal cancer and its inhibitory effect on the tumor growth in vitro and in vivo Int J Cancer 2009 125 1505 1513 10.1002/ijc.24513 19521989

[B15] Li W Liu X Zhang B Qi D Zhang L Jin Y Yang H Overexpression of candidate tumor suppressor ECRG4 inhibits Gliomas proliferation and invasion J Exp Clin Cancer Res 2010 29 89 10.1186/1756-9966-29-89 20598162 PMC2913949

[B16] Yue CM Deng DJ Bi MX Guo LP Lu SH Expression of ECRG4, a novel esophageal cancer-related gene, downregulated by CpG island hypermethylation in human esophageal squamous cell carcinoma World J Gastroenterol 2003 9 1174 1178 12800218 10.3748/wjg.v9.i6.1174 PMC4611778

[B17] Mori Y Ishiguro H Kuwabara Y Kimura M Mitsui A Kurehara H Mori R Tomoda K Ogawa R Katada T Harata K Fujii Y Expression of ECRG4 is an independent prognostic factor for poor survival in patients with esophageal squamous cell carcinoma Oncol Rep 2007 18 981 985 17786363

[B18] Vanaja DK Ehrich M Van den Boom D Cheville JC Karnes RJ Tindall DJ Cantor CR Young CY Hypermethylation of genes for diagnosis and risk stratification of prostate cancer Cancer Invest 2009 27 549 560 10.1080/07357900802620794 19229700 PMC2693083

[B19] Kujuro Y Suzuki N Kondo T Esophageal cancer-related gene 4 is a secreted inducer of cell senescence expressed by aged CNS precursor cells Proc Natl Acad Sci USA 2010 107 8259 8264 10.1073/pnas.0911446107 20404145 PMC2889548

[B20] Magdaleno S Jensen P Brumwell CL Seal A Lehman K Asbury A Cheung T Cornelius T Batten DM Eden C Norland SM Dosooye N Shakya S Mehta P Curran T BGEM: an in situ hybridization database of gene expression in the embryonic and adult mouse nervous system PLoS Biol 2006 4 e86 10.1371/journal.pbio.0040086 16602821 PMC1413568

[B21] Allen Brain Atlas Resources [Internet] 2009 Seattle (WA): Allen Institute for Brain Science http://www.brain-map.org

[B22] International Knockout Mouse Consortium http://www.knockoutmouse.org/genedetails/MGI:1926146

[B23] Maxwell WL Follows R Ashhurst DE Berry M The response of the cerebral hemisphere of the rat to injury. I. The mature rat Philos Trans R Soc Lond B Biol Sci 1990 328 479 500 10.1098/rstb.1990.0121 1974074

[B24] Logan A Green J Hunter A Jackson R Berry M Inhibition of glial scarring in the injured rat brain by a recombinant human monoclonal antibody to transforming growth factor-beta2 Eur J Neurosci 1999 11 2367 2374 10.1046/j.1460-9568.1999.00654.x 10383626

[B25] Leadbeater WE Gonzalez AM Logaras N Berry M Turnbull JE Logan A Intracellular trafficking in neurones and glia of fibroblast growth factor-2, fibroblast growth factor receptor 1 and heparan sulphate proteoglycans in the injured adult rat cerebral cortex J Neurochem 2006 96 1189 1200 10.1111/j.1471-4159.2005.03632.x 16417571

[B26] Johanson CE Szmydynger-Chodobska J Chodobski A Baird A McMillan P Stopa EG Altered formation and bulk absorption of cerebrospinal fluid in FGF-2-induced hydrocephalus Am J Physiol 1999 277 R263 271 10409281 10.1152/ajpregu.1999.277.1.R263

[B27] Shan Q Storm DR Optimization of a cAMP response element signal pathway reporter system J Neurosci Methods 2010 10.1016/j.jneumeth.2010.06.003 PMC3506255 20540964

[B28] Huh YH Ryu JH Shin S Lee DU Yang S Oh KS Chun CH Choi JK Song WK Chun JS Esophageal cancer related gene 4 (ECRG4) is a marker of articular chondrocyte differentiation and cartilage destruction Gene 2009 448 7 15 10.1016/j.gene.2009.08.015 19735703

[B29] Engelhardt B Wolburg-Buchholz K Wolburg H Involvement of the choroid plexus in central nervous system inflammation Microsc Res Tech 2001 52 112 129 10.1002/1097-0029(20010101)52:1<112::AID-JEMT13>3.0.CO;2-5 11135454

[B30] Ennis SR Keep RF Forebrain ischemia and the blood-cerebrospinal fluid barrier Acta Neurochir Suppl 2006 96 276 278 full_text 16671470 10.1007/3-211-30714-1_59

[B31] Zhang Y Bergelson JM Adenovirus receptors J Virol 2005 79 12125 12131 10.1128/JVI.79.19.12125-12131.2005 16160140 PMC1211528

[B32] Herenu CB Sonntag WE Morel GR Portiansky EL Goya RG The ependymal route for insulin-like growth factor-1 gene therapy in the brain Neuroscience 2009 163 442 447 10.1016/j.neuroscience.2009.06.024 19531373 PMC2740751

[B33] Mokry J Karbanova J Osterreicher J Experimental brain injury induces activation of neural stem cells in the forebrain subependyma Appl Immunohistochem Mol Morphol 2003 11 161 167 12778002 10.1097/00129039-200306000-00013

[B34] Holmin S Almqvist P Lendahl U Mathiesen T Adult nestin-expressing subependymal cells differentiate to astrocytes in response to brain injury Eur J Neurosci 1997 9 65 75 10.1111/j.1460-9568.1997.tb01354.x 9042570

[B35] Velocigene http://www.velocigene.com/komp/detail/10814

[B36] The Sanger Institute http://www.sanger.ac.uk/htgt/report/gene_report?project_id=23064

[B37] Seabra R Bhogal N In vivo research using early life stage models In Vivo 2010 24 457 462 20668311

[B38] Obulesu M Rao DM Animal models of Alzheimer's disease: an understanding of pathology and therapeutic avenues Int J Neurosci 2010 120 531 537 10.3109/00207451003760080 20615056

[B39] Peal DS Peterson RT Milan D Small Molecule Screening in Zebrafish J Cardiovasc Transl Res 2010 3 454 460 10.1007/s12265-010-9212-8 20680709

[B40] Garcia-Lecea M Kondrychyn I Fong SH Ye ZR Korzh V In vivo analysis of choroid plexus morphogenesis in zebrafish PLoS One 2008 3 e3090 10.1371/journal.pone.0003090 18769618 PMC2525818

[B41] Zupanc GK Wellbrock UM Sirbulescu RF Rajendran RS Generation, long-term persistence, and neuronal differentiation of cells with nuclear aberrations in the adult zebrafish brain Neuroscience 2009 159 1338 1348 10.1016/j.neuroscience.2009.02.014 19217927

[B42] Chodobski A Szmydynger-Chodobska J Choroid plexus: target for polypeptides and site of their synthesis Microsc Res Tech 2001 52 65 82 10.1002/1097-0029(20010101)52:1<65::AID-JEMT9>3.0.CO;2-4 11135450

[B43] Skipor J Thiery JC The choroid plexus--cerebrospinal fluid system: undervaluated pathway of neuroendocrine signaling into the brain Acta Neurobiol Exp (Wars) 2008 68 414 428 18668165 10.55782/ane-2008-1708

[B44] Roberton AGA Stopa E Leadbeater W Coimbra R Johanson CE Eliceiri B Baird A Immunohistochemical evidence that argilin, the product of the ECRG4 gene, encodes a novel neuroendocrine peptide Endocrine Abstracts, Society for Endocrinology BES 2009, Harrogate, UK 2009 15 S82

[B45] Podvin SGA Roberton A Johanson C Stopa E Eliceiri BP Baird A Augrin, Ecilin and Argilin: Characterization of neuropeptide candidates encoded by the esophageal cancer related Gene-4 (ecrg4) and their localization in the mouse choroid plexus 2009 Society for Neuroscience Planner 85.88/CC32

[B46] Tadross JA Patterson M Suzuki K Beale KE Boughton CK Smith KL Moore S Ghatei MA Bloom SR Augurin stimulates the hypothalamo-pituitary-adrenal axis via the release of corticotrophin-releasing factor in rats Br J Pharmacol 2010 159 1663 1671 10.1111/j.1476-5381.2010.00655.x 20233222 PMC2925489

[B47] Klein R Bidirectional modulation of synaptic functions by Eph/ephrin signaling Nat Neurosci 2009 12 15 20 10.1038/nn.2231 19029886

[B48] Lathia JD Mattson MP Cheng A Notch: from neural development to neurological disorders J Neurochem 2008 107 1471 1481 10.1111/j.1471-4159.2008.05715.x 19094054 PMC4544712

[B49] Nishikawa M Drmanac R Radoje T Lobal I Tang T Lee J Stache-Crain B Polypeptide having an activity to support proliferation or survival of hematopoietic stem cell and hematopoietic progenitor cell, and DNA coding for the same US Patent 7320880 2008

[B50] Donahue JEMM Breeze V Podvin S Eliceiri B Jackson CL Johanson CE Stopa E Gonzalez AM Baird A Ecrg4 in Normal and Neoplastic Choroid Plexus Society for Research into Hydrocephalus and Spina Bifida, Vancouver, BC, Canada 2010

[B51] Podvin SGA Botfield H Leadbeater W Dang X Hill L Boissaud-Cooke M Knowling S Logan A Bansal V Coimbra R Stopa E Johanson C Eliceiri BP Baird A The choroid plexus response to CNS injury: Augurin, a secreted ligand encoded by the tumor suppressor gene, ecrg4 Society for Neuroscience Planner 2010 4 902.910/JJJ912

